# Bis[*N*-benzyl-*N*-(2-phenyl­eth­yl)dithio­carbamato-κ^2^ *S*,*S*′]lead(II)

**DOI:** 10.1107/S1600536812036161

**Published:** 2012-08-25

**Authors:** E. Sathiyaraj, S. Thirumaran, S. Selvanayagam

**Affiliations:** a Department of Chemistry, Annamalai University, Annamalainagar 608 002, India; b Department of Physics, Kalasalingam University, Krishnankoil 626 126, India

## Abstract

The mol­ecule of the title compound, [Pb(C_16_H_16_NS_2_)_2_], is located on a twofold rotation axis, which runs through the Pb^II^ atom. The two dithio­carbamate ligands coordinate the metal in a pyramidal configuration through the S atoms. The two phenyl rings of each dithocarbamate ligand are aligned at a dihedral angle of 78.4 (1)°. The mol­ecular conformation is stabilized by intra­molecular C—H⋯S inter­actions.

## Related literature

For general background of the title compound, see: Davidovich *et al.* (2010 ▶); Picket & O’Brien (2001 ▶); Srinivasan & Thirumaran (2012 ▶); Sathiyaraj & Thirumaran (2012 ▶); Green *et al.* (2004 ▶); Koh *et al.* (2003 ▶). For the preparation, see: Sathiyaraj & Thirumaran (2012 ▶). For a related structure, see: Davidovich *et al.* (2010 ▶)
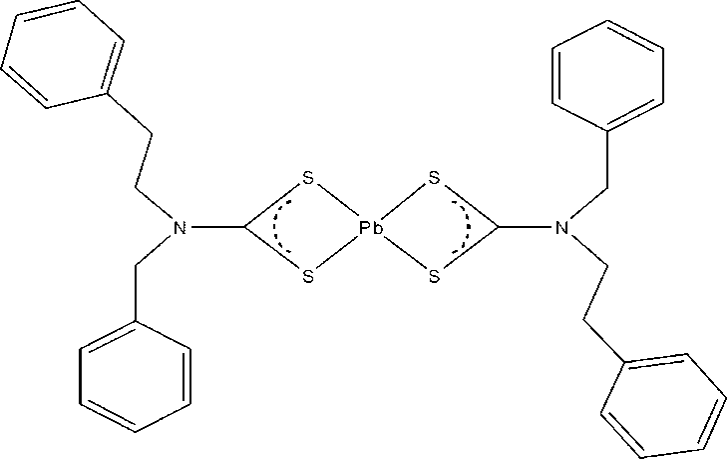


## Experimental

### 

#### Crystal data

[Pb(C_16_H_16_NS_2_)_2_]
*M* *_r_* = 780.03
Monoclinic, 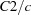

*a* = 28.5467 (12) Å
*b* = 5.5321 (2) Å
*c* = 19.4158 (8) Å
β = 101.600 (2)°
*V* = 3003.6 (2) Å^3^
*Z* = 4
Mo *K*α radiation
μ = 5.92 mm^−1^
*T* = 292 K
0.20 × 0.20 × 0.20 mm

#### Data collection

Bruker SMART APEXII area-detector diffractometer
Absorption correction: multi-scan (*SADABS*; Bruker, 2008 ▶) *T*_min_ = 0.384, *T*_max_ = 0.384
13027 measured reflections
3711 independent reflections
3006 reflections with *I* > 2σ(*I*)
*R*_int_ = 0.038

#### Refinement

*R*[*F* ^2^ > 2σ(*F* ^2^)] = 0.028
*wR*(*F* ^2^) = 0.062
*S* = 1.04
3711 reflections
177 parameters
H-atom parameters constrained
Δρ_max_ = 0.43 e Å^−3^
Δρ_min_ = −1.25 e Å^−3^

### 

Data collection: *APEX2* (Bruker, 2008 ▶); cell refinement: *SAINT* (Bruker, 2008 ▶); data reduction: *SAINT*; program(s) used to solve structure: *SIR92* (Altomare *et al.*, 1993 ▶); program(s) used to refine structure: *SHELXL97* (Sheldrick, 2008 ▶); molecular graphics: *ORTEP-3* (Farrugia, 1997 ▶) and *PLATON* (Spek, 2009 ▶); software used to prepare material for publication: *SHELXL97* and *PLATON*.

## Supplementary Material

Crystal structure: contains datablock(s) I, global. DOI: 10.1107/S1600536812036161/bt5972sup1.cif

Structure factors: contains datablock(s) I. DOI: 10.1107/S1600536812036161/bt5972Isup2.hkl

Additional supplementary materials:  crystallographic information; 3D view; checkCIF report

## Figures and Tables

**Table 1:** Hydrogen-bond geometry (Å, °)

*D*—H⋯*A*	*D*—H	H⋯*A*	*D*⋯*A*	*D*—H⋯*A*
C9—H9*A*⋯S2	0.97	2.49	2.986 (3)	112
C2—H2*B*⋯S1	0.97	2.55	2.990 (4)	107

**Fig. 1.**
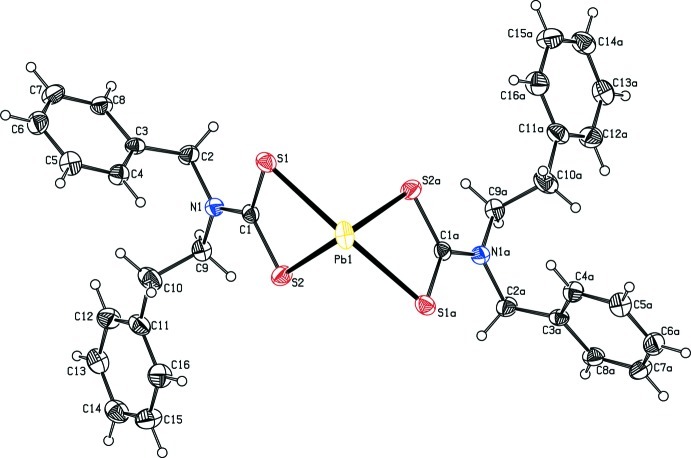
The molecular structure of the title compound, showing the atom-numbering scheme. Displacement ellipsoids are drawn at the 30% probability level.

**Fig. 2.**
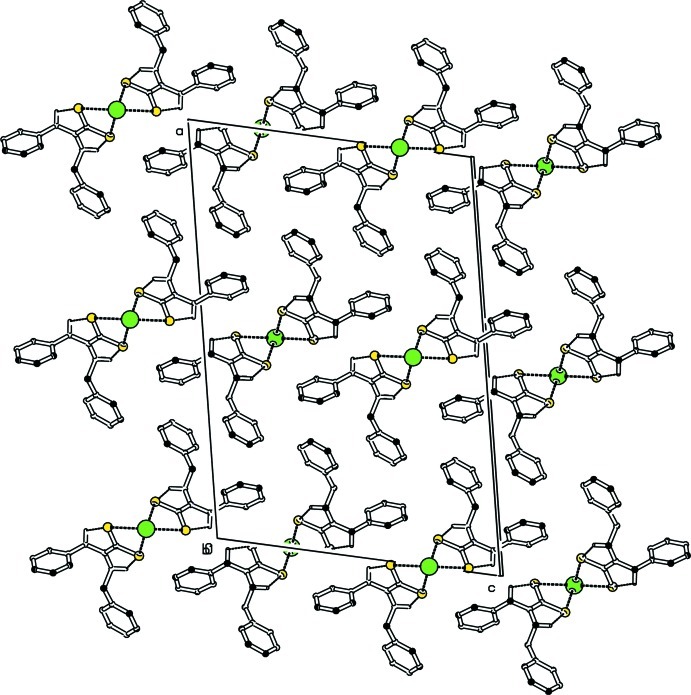
Molecular packing of the title compound, viewed along the b axis; H-bonds are shown as dashed lines. For the sake of clarity, H atoms, not involved in hydrogen bonds, have been omitted.

## supplementary crystallographic information

### Comment

The solid state structural chemistry of lead dithiocarbamates is rich and fascinating in that different structural motifs are found ranging from monomeric, dimeric, tetrameric to linear chain (Davidovich *et al.*, 2010). Metal dithiocarbamate complexes have proven to be very successful as single source precursors for the preparation of metal sulfide nanoparticles (Picket & O'Brien, 2001; Srinivasan & Thirumaran, 2012). The title compound was also used as single source precursor for the synthesis of PbS nanoparticles (Sathiyaraj & Thirumaran, 2012). There is an indication that the molecular structure of the synthetic precursor may influence both the size and morphology of the nanoparticles (Green *et al.*, 2004; Koh *et al.*, 2003). In view of these importance we have undertaken the crystal structure determination of the title compound, and the results are presented here.

The X-ray study confirmed the molecular structure and atomic connectivity for (I), as illustrated in Fig. 1.

The structure consists of monomeric molecules composed of one Pb atom and two chelating dithiocarbamate ligands. The two dithiocarbamate ligands are coordinated through S atoms to the metal pyramidally and in each chelate ring one Pb-S bond is significantly shorter than other. The relative bond distances and angles for the title compound agree with the presence of an electron lone pair at an equatorial position of a distorted trigonal bipyramid PbS_4_. Evidence for the presence of a stereochemically active electron lone pair of the lead atom has also been reported for other lead complexes (Davidovich *et al.*, 2010).

The sum of the angles at N1 [359.8°] is in accordance with sp^2^ hybridization. Two phenyl rings in dithiocarbmate ligand is make a dihedral angle of 78.4 (1) °.

In addition to the van der Waals interactions, the molecular structure is influenced only by intramolecular C—H···S hydrogen bonds involving atoms S1 and S2. (Fig. 2 and Table 1).

### Experimental

The title compound was prepared according to the literature procedure (Sathiyaraj & Thirumaran, 2012). Single crystals were obtained by slow evaporation of dichloromethane and acetone (1:1) solution of the title compound at room temperature.

### Refinement

H atoms were placed in idealized positions and allowed to ride on their parent atoms, with C—H distances of 0.93-0.97 Å, and Uiso(H) = 1.2U_eq_(C) for H atoms.

### Crystal data

**Table:**

[Pb(C_16_H_16_NS_2_)_2_]	*F*(000) = 1536
*M**_r_* = 780.03	*D*_x_ = 1.725 Mg m^−^^3^
Monoclinic, *C*2/*c*	Mo *K*α radiation, λ = 0.71073 Å
Hall symbol: -C 2yc	Cell parameters from 5527 reflections
*a* = 28.5467 (12) Å	θ = 1.5–28.3°
*b* = 5.5321 (2) Å	µ = 5.92 mm^−^^1^
*c* = 19.4158 (8) Å	*T* = 292 K
β = 101.600 (2)°	Block, brown
*V* = 3003.6 (2) Å^3^	0.20 × 0.20 × 0.20 mm
*Z* = 4	

### Data collection

**Table:**

Bruker SMART APEXII area-detector diffractometer	3711 independent reflections
Radiation source: fine-focus sealed tube	3006 reflections with *I* > 2σ(*I*)
Graphite monochromator	*R*_int_ = 0.038
ω and φ scans	θ_max_ = 28.3°, θ_min_ = 1.5°
Absorption correction: multi-scan (*SADABS*; Bruker, 2008)	*h* = −37→31
*T*_min_ = 0.384, *T*_max_ = 0.384	*k* = −7→7
13027 measured reflections	*l* = −25→25

### Refinement

**Table:**

Refinement on *F*^2^	Primary atom site location: structure-invariant direct methods
Least-squares matrix: full	Secondary atom site location: difference Fourier map
*R*[*F*^2^ > 2σ(*F*^2^)] = 0.028	Hydrogen site location: inferred from neighbouring sites
*wR*(*F*^2^) = 0.062	H-atom parameters constrained
*S* = 1.04	*w* = 1/[σ^2^(*F*_o_^2^) + (0.0254*P*)^2^ + 1.657*P*] where *P* = (*F*_o_^2^ + 2*F*_c_^2^)/3
3711 reflections	(Δ/σ)_max_ = 0.001
177 parameters	Δρ_max_ = 0.43 e Å^−^^3^
0 restraints	Δρ_min_ = −1.25 e Å^−^^3^

### Special details

**Table:**

Geometry. All esds (except the esd in the dihedral angle between two l.s. planes) are estimated using the full covariance matrix. The cell esds are taken into account individually in the estimation of esds in distances, angles and torsion angles; correlations between esds in cell parameters are only used when they are defined by crystal symmetry. An approximate (isotropic) treatment of cell esds is used for estimating esds involving l.s. planes.
Refinement. Refinement of F^2^ against ALL reflections. The weighted R-factor wR and goodness of fit S are based on F^2^, conventional R-factors R are based on F, with F set to zero for negative F^2^. The threshold expression of F^2^ > 2sigma(F^2^) is used only for calculating R-factors(gt) etc. and is not relevant to the choice of reflections for refinement. R-factors based on F^2^ are statistically about twice as large as those based on F, and R- factors based on ALL data will be even larger.

### Fractional atomic coordinates and isotropic or equivalent isotropic displacement parameters (Å^2^)

**Table:**

	*x*	*y*	*z*	*U*_iso_*/*U*_eq_	
Pb1	1.0000	1.14280 (3)	0.7500	0.05084 (8)	
S1	0.99115 (3)	0.94809 (17)	0.61154 (4)	0.04494 (19)	
S2	0.93213 (3)	0.80487 (16)	0.71424 (4)	0.0473 (2)	
N1	0.92789 (9)	0.5899 (4)	0.59107 (12)	0.0373 (6)	
C1	0.94871 (10)	0.7651 (6)	0.63419 (14)	0.0339 (6)	
C2	0.94211 (11)	0.5417 (6)	0.52408 (15)	0.0440 (7)	
H2A	0.9397	0.3691	0.5153	0.053*	
H2B	0.9755	0.5862	0.5287	0.053*	
C3	0.91358 (11)	0.6706 (5)	0.46061 (15)	0.0343 (6)	
C4	0.88722 (12)	0.8758 (5)	0.46490 (17)	0.0427 (7)	
H4	0.8853	0.9369	0.5089	0.051*	
C5	0.86360 (12)	0.9926 (6)	0.40547 (17)	0.0498 (8)	
H5	0.8460	1.1317	0.4095	0.060*	
C6	0.86593 (13)	0.9041 (7)	0.34018 (18)	0.0571 (10)	
H6	0.8501	0.9832	0.2999	0.069*	
C7	0.89154 (14)	0.6993 (8)	0.33484 (17)	0.0569 (10)	
H7	0.8931	0.6391	0.2906	0.068*	
C8	0.91528 (12)	0.5799 (6)	0.39433 (16)	0.0453 (8)	
H8	0.9323	0.4393	0.3900	0.054*	
C9	0.89245 (11)	0.4222 (6)	0.61033 (17)	0.0419 (7)	
H9A	0.8957	0.4225	0.6610	0.050*	
H9B	0.8992	0.2598	0.5962	0.050*	
C10	0.84215 (12)	0.4865 (8)	0.5771 (2)	0.0612 (10)	
H10A	0.8382	0.4756	0.5264	0.073*	
H10B	0.8360	0.6523	0.5888	0.073*	
C11	0.80629 (12)	0.3225 (6)	0.60110 (19)	0.0492 (8)	
C12	0.78865 (14)	0.1181 (7)	0.5636 (2)	0.0564 (9)	
H12	0.8000	0.0762	0.5236	0.068*	
C13	0.75473 (12)	−0.0232 (7)	0.58444 (19)	0.0542 (9)	
H13	0.7434	−0.1599	0.5585	0.065*	
C14	0.73735 (13)	0.0338 (7)	0.6428 (2)	0.0579 (9)	
H14	0.7137	−0.0608	0.6561	0.070*	
C15	0.75511 (14)	0.2325 (8)	0.68166 (19)	0.0629 (10)	
H15	0.7439	0.2719	0.7220	0.075*	
C16	0.78937 (14)	0.3730 (6)	0.6612 (2)	0.0584 (10)	
H16	0.8015	0.5057	0.6885	0.070*	

### Atomic displacement parameters (Å^2^)

**Table:**

	*U*^11^	*U*^22^	*U*^33^	*U*^12^	*U*^13^	*U*^23^
Pb1	0.05114 (13)	0.03239 (11)	0.05977 (12)	0.000	−0.01087 (8)	0.000
S1	0.0350 (4)	0.0494 (5)	0.0505 (4)	−0.0041 (4)	0.0088 (3)	0.0104 (4)
S2	0.0515 (5)	0.0542 (5)	0.0374 (4)	−0.0131 (4)	0.0120 (4)	−0.0068 (4)
N1	0.0362 (14)	0.0397 (14)	0.0366 (12)	−0.0003 (11)	0.0088 (11)	−0.0024 (11)
C1	0.0303 (15)	0.0344 (15)	0.0355 (14)	0.0031 (13)	0.0033 (12)	0.0036 (13)
C2	0.0473 (19)	0.0463 (18)	0.0398 (15)	0.0094 (16)	0.0121 (14)	−0.0061 (15)
C3	0.0352 (16)	0.0323 (16)	0.0381 (14)	−0.0058 (12)	0.0142 (13)	−0.0051 (12)
C4	0.0476 (19)	0.0378 (18)	0.0431 (16)	−0.0015 (14)	0.0100 (14)	−0.0079 (14)
C5	0.054 (2)	0.0366 (19)	0.0565 (19)	0.0008 (15)	0.0067 (16)	0.0025 (16)
C6	0.055 (2)	0.068 (3)	0.0470 (19)	−0.0091 (19)	0.0068 (17)	0.0178 (18)
C7	0.062 (2)	0.075 (3)	0.0374 (17)	−0.008 (2)	0.0185 (17)	−0.0050 (17)
C8	0.0453 (19)	0.0483 (19)	0.0460 (17)	−0.0039 (15)	0.0182 (15)	−0.0098 (15)
C9	0.0423 (18)	0.0355 (16)	0.0459 (16)	0.0000 (14)	0.0045 (14)	−0.0028 (14)
C10	0.041 (2)	0.067 (3)	0.076 (2)	0.0087 (18)	0.0135 (18)	0.030 (2)
C11	0.0356 (18)	0.052 (2)	0.061 (2)	0.0064 (15)	0.0116 (16)	0.0140 (17)
C12	0.052 (2)	0.063 (3)	0.057 (2)	0.0107 (18)	0.0173 (18)	−0.0014 (19)
C13	0.049 (2)	0.043 (2)	0.066 (2)	−0.0022 (16)	0.0033 (17)	−0.0008 (18)
C14	0.047 (2)	0.057 (2)	0.071 (2)	−0.0051 (18)	0.0141 (19)	0.016 (2)
C15	0.069 (3)	0.069 (3)	0.058 (2)	−0.007 (2)	0.029 (2)	−0.001 (2)
C16	0.058 (2)	0.054 (2)	0.063 (2)	−0.0103 (17)	0.0127 (19)	−0.0096 (17)

### Geometric parameters (Å, º)

**Table:**

Pb1—S2^i^	2.6813 (8)	C7—C8	1.384 (5)
Pb1—S2	2.6813 (8)	C7—H7	0.9300
Pb1—S1^i^	2.8597 (9)	C8—H8	0.9300
Pb1—S1	2.8597 (9)	C9—C10	1.494 (4)
S1—C1	1.703 (3)	C9—H9A	0.9700
S2—C1	1.727 (3)	C9—H9B	0.9700
N1—C1	1.339 (4)	C10—C11	1.510 (5)
N1—C2	1.463 (4)	C10—H10A	0.9700
N1—C9	1.475 (4)	C10—H10B	0.9700
C2—C3	1.512 (4)	C11—C16	1.378 (5)
C2—H2A	0.9700	C11—C12	1.384 (5)
C2—H2B	0.9700	C12—C13	1.368 (5)
C3—C4	1.373 (4)	C12—H12	0.9300
C3—C8	1.391 (4)	C13—C14	1.362 (5)
C4—C5	1.375 (4)	C13—H13	0.9300
C4—H4	0.9300	C14—C15	1.372 (6)
C5—C6	1.373 (5)	C14—H14	0.9300
C5—H5	0.9300	C15—C16	1.369 (5)
C6—C7	1.363 (5)	C15—H15	0.9300
C6—H6	0.9300	C16—H16	0.9300
			
S2^i^—Pb1—S2	91.59 (4)	C8—C7—H7	119.6
S2^i^—Pb1—S1^i^	64.61 (2)	C7—C8—C3	119.8 (3)
S2—Pb1—S1^i^	84.46 (2)	C7—C8—H8	120.1
S2^i^—Pb1—S1	84.46 (2)	C3—C8—H8	120.1
S2—Pb1—S1	64.61 (2)	N1—C9—C10	112.9 (3)
S1^i^—Pb1—S1	135.74 (4)	N1—C9—H9A	109.0
C1—S1—Pb1	85.08 (10)	C10—C9—H9A	109.0
C1—S2—Pb1	90.43 (11)	N1—C9—H9B	109.0
C1—N1—C2	121.3 (3)	C10—C9—H9B	109.0
C1—N1—C9	122.5 (2)	H9A—C9—H9B	107.8
C2—N1—C9	116.0 (2)	C9—C10—C11	112.1 (3)
N1—C1—S1	121.2 (2)	C9—C10—H10A	109.2
N1—C1—S2	119.2 (2)	C11—C10—H10A	109.2
S1—C1—S2	119.63 (17)	C9—C10—H10B	109.2
N1—C2—C3	116.0 (2)	C11—C10—H10B	109.2
N1—C2—H2A	108.3	H10A—C10—H10B	107.9
C3—C2—H2A	108.3	C16—C11—C12	117.3 (3)
N1—C2—H2B	108.3	C16—C11—C10	121.0 (3)
C3—C2—H2B	108.3	C12—C11—C10	121.7 (3)
H2A—C2—H2B	107.4	C13—C12—C11	121.1 (3)
C4—C3—C8	118.4 (3)	C13—C12—H12	119.5
C4—C3—C2	123.5 (3)	C11—C12—H12	119.5
C8—C3—C2	118.1 (3)	C14—C13—C12	120.8 (3)
C3—C4—C5	121.3 (3)	C14—C13—H13	119.6
C3—C4—H4	119.3	C12—C13—H13	119.6
C5—C4—H4	119.3	C13—C14—C15	119.2 (3)
C6—C5—C4	120.0 (3)	C13—C14—H14	120.4
C6—C5—H5	120.0	C15—C14—H14	120.4
C4—C5—H5	120.0	C16—C15—C14	120.1 (3)
C7—C6—C5	119.5 (3)	C16—C15—H15	119.9
C7—C6—H6	120.2	C14—C15—H15	119.9
C5—C6—H6	120.2	C15—C16—C11	121.5 (3)
C6—C7—C8	120.9 (3)	C15—C16—H16	119.2
C6—C7—H7	119.6	C11—C16—H16	119.2
			
S2^i^—Pb1—S1—C1	91.43 (10)	C3—C4—C5—C6	0.3 (5)
S2—Pb1—S1—C1	−2.98 (10)	C4—C5—C6—C7	0.4 (5)
S1^i^—Pb1—S1—C1	47.21 (10)	C5—C6—C7—C8	−0.1 (6)
S2^i^—Pb1—S2—C1	−80.18 (10)	C6—C7—C8—C3	−0.8 (5)
S1^i^—Pb1—S2—C1	−144.48 (10)	C4—C3—C8—C7	1.4 (5)
S1—Pb1—S2—C1	2.93 (10)	C2—C3—C8—C7	−176.9 (3)
C2—N1—C1—S1	2.6 (4)	C1—N1—C9—C10	101.6 (3)
C9—N1—C1—S1	177.5 (2)	C2—N1—C9—C10	−83.3 (3)
C2—N1—C1—S2	−177.3 (2)	N1—C9—C10—C11	−176.3 (3)
C9—N1—C1—S2	−2.4 (4)	C9—C10—C11—C16	87.0 (4)
Pb1—S1—C1—N1	−175.1 (2)	C9—C10—C11—C12	−93.8 (4)
Pb1—S1—C1—S2	4.81 (16)	C16—C11—C12—C13	1.9 (5)
Pb1—S2—C1—N1	174.8 (2)	C10—C11—C12—C13	−177.3 (3)
Pb1—S2—C1—S1	−5.11 (17)	C11—C12—C13—C14	0.1 (5)
C1—N1—C2—C3	−92.9 (3)	C12—C13—C14—C15	−1.5 (6)
C9—N1—C2—C3	91.9 (3)	C13—C14—C15—C16	1.0 (6)
N1—C2—C3—C4	20.9 (5)	C14—C15—C16—C11	1.1 (6)
N1—C2—C3—C8	−160.9 (3)	C12—C11—C16—C15	−2.5 (5)
C8—C3—C4—C5	−1.1 (5)	C10—C11—C16—C15	176.7 (4)
C2—C3—C4—C5	177.0 (3)		

Symmetry code: (i) −*x*+2, *y*, −*z*+3/2.

### Hydrogen-bond geometry (Å, º)

**Table:**

*D*—H···*A*	*D*—H	H···*A*	*D*···*A*	*D*—H···*A*
C9—H9*A*···S2	0.97	2.49	2.986 (3)	112
C2—H2*B*···S1	0.97	2.55	2.990 (4)	107
